# Reversing stage III oral adenocarcinoma in a dog treated with anti-canine PD-1 therapeutic antibody: a case report

**DOI:** 10.3389/fvets.2023.1144869

**Published:** 2023-05-11

**Authors:** Shuo Xu, Jingshu Xie, Shuaiyu Wang, Na Tang, Junli Feng, Youhong Su, Gebin Li

**Affiliations:** ^1^College of Veterinary Medicine, China Agricultural University, Beijing, China; ^2^Biocytogen Pharmaceuticals (Beijing) Co., Ltd., Beijing, China

**Keywords:** canine, salivary gland carcinoma, immunotherapy, monoclonal antibody, programmed cell death receptor 1, partial remission

## Abstract

Monoclonal antibody targeting programmed cell death-1 (PD-1) is one of the most promising treatment therapies for human cancers. Canine PD-1 antibodies used in clinical trials have also shown efficacy in treating canine cancers. An 11-year-old male intact border collie presented to us for evaluation of left cervical mass. Computed tomography (CT) examination revealed an irregular pharyngeal mass invading the surrounding soft tissue. Histological and immunohistochemical results were consistent with a diagnosis of adenocarcinoma, most likely originating from the minor salivary gland. An anti-canine PD-1 monoclonal antibody was administered. Two months after the initial treatment, the tumor reached partial remission and maintained as such for 6 months. Finally, the patient was euthanized due to reasons unrelated to cancer, with a survival time of 316 days. To our knowledge, this is the first report of response to PD-1 blockade treatment in canine adenocarcinoma.

## 1. Introduction

Salivary gland neoplasms are primarily malignant epithelial tumors and rare in dogs. The incidence of salivary neoplasia in this population was calculated to be 15.3 per 100,000 dogs, which may be overestimated (1). In one retrospective study of salivary gland tumors in dogs and cats, only 3 of 24 cases originated from sublingual and minor glands, suggesting that minor gland neoplasia is much rarer (2). Surgery is the mainstay of therapy. Even though chemotherapy is recommended it is not frequently described due to the poor efficacy and the paucity of information in the literature. There are currently no particularly effective treatment options, especially for tumors unsuitable for excisional surgery.

Immune checkpoint inhibitors (ICIs) have become attractive treatment options for a wide range of cancers in human. The first ICI, an antibody targeting the cytotoxic T lymphocyte antigen 4 (Ipilimumab), was approved by the Food and Drug Administration (FDA) to treat metastatic melanoma in 2011 (3). Programmed cell death receptor-1 (PD-1) and its ligands (PD-L1 and PD-L2) are one of the most attractive immune checkpoints. PD-1 can be expressed in activated T and B cells, macrophages and NK cells, while PD-L1 can be widely expressed in non-lymphoid tissues, including tumor cells and tumor infiltrating immune cells (4–6). Since the PD-1 signaling pathway inhibits T cell proliferation and cytokine production, PD-1 blockade reverses PD-1-mediated anti-tumor immunosuppression (7, 8). A number of PD-1 and PD-L1 inhibitors have been developed and approved for anti-cancer therapy in humans. Up to now, three anti-PD-1 antibodies have been approved by the FDA: pembrolizumab (Keytruda), nivolumab (Opdivo), and cemiplimab (Libtayo), they can be used to treat human cancer including unresectable or metastatic melanoma, non-small cell lung cancer, and squamous cell carcinoma (9). In veterinary medicine, PD-1/PD-L1 antibody has been used in the treatment of oral malignant melanoma (OMM) and achieved certain efficacy (10, 11), but all of them are limited to clinical trials and none has been approved for anti-cancer therapy of dogs.

In this case, the patient with oral adenocarcinoma was treated with the anti-canine PD-1 monoclonal antibody named MP001, produced by Biocytogen Pharmaceuticals (Beijing) Co., Ltd. MP001 is a fully caninized antibody and the immunoglobulin concentration is 10 mg/mL. To our knowledge, treatment with PD-1 blockade in canine oral adenocarcinoma has not been reported previously.

## 2. Case presentation

An 11-year-old, male intact border collie was presented to the China Agricultural University veterinary teaching hospital with a left cervical mass. The mass has been present for two weeks with clinical symptoms such as ptyalism, dysphagia and occasionally tachypnea.

On physical examination, 4/9 body condition score, normal mental state, adequate hydration, pink mucosa, normal capillary refill time and normal rectal temperature were detected. Except for the mass, there was no obvious enlargement of superficial lymph nodes (LNs). On palpation, the cervical mass was located deep on the left ventral side, solid and fixed. No obvious mass could be detected on oral examination without anesthesia.

The mass was further identified by cervical ultrasound examination, showing mixed echogenic and cavitated appearance on the left neck with mild vascularity, measuring approximately 3.34 × 2.87 cm on the sagittal plane. The left medial retropharyngeal LN was unevenly hypoechoic and poorly demarcated from the mass. Soft tissue in the pharynx was hypoechoic of up to 1.0 cm thickness. In addition, CBC and serum biochemistry were within normal limits. No abnormalities were detected via two-view thoracic radiographs.

**Figure 1:**
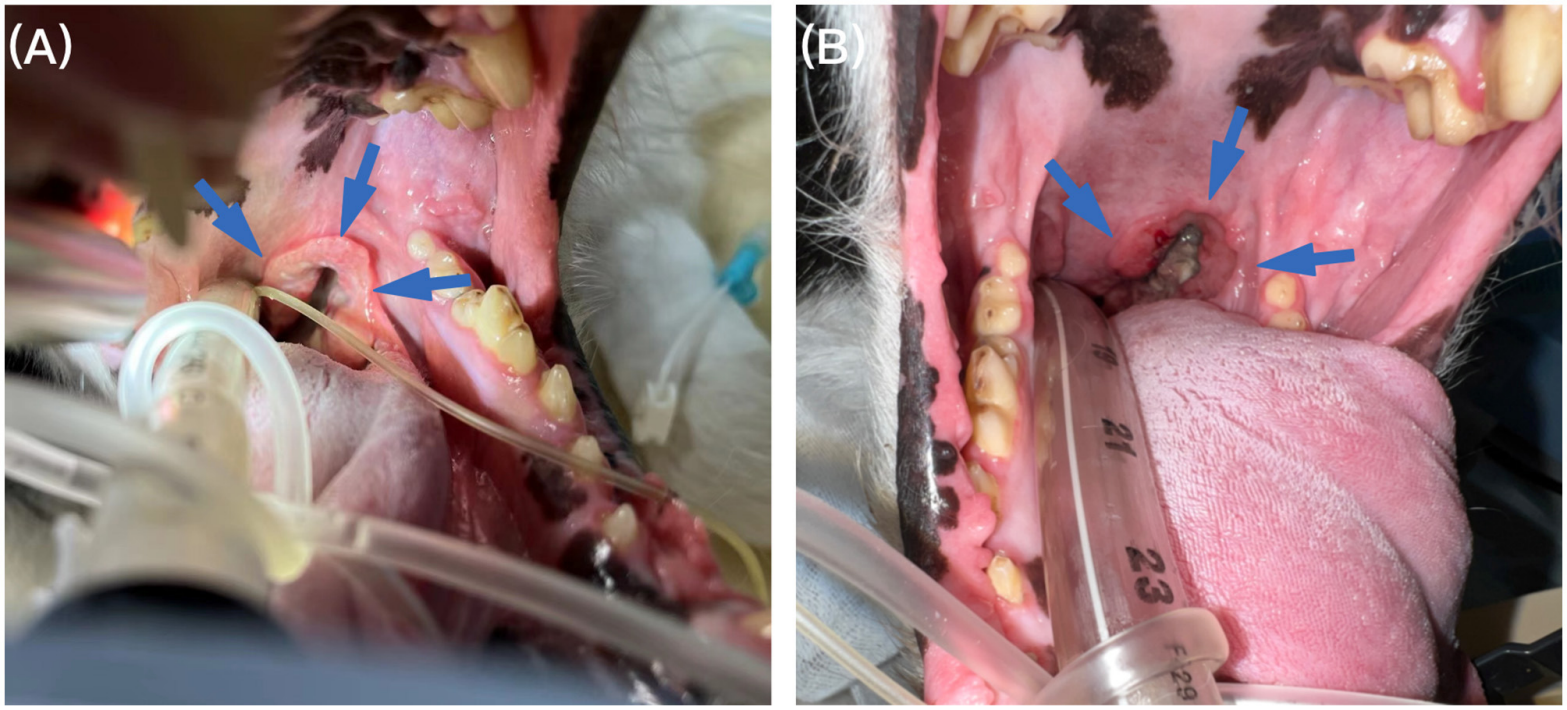
Oral examination under anesthesia. **(A)** Oral examination under anesthesia revealed a ring-thickened mass on the left soft palate (blue arrow) before treatment. **(B)** Follow-up 4 months, the soft palate mass (blue arrow) was significantly reduced on oral examination after anesthesia.

**Figure 2:**
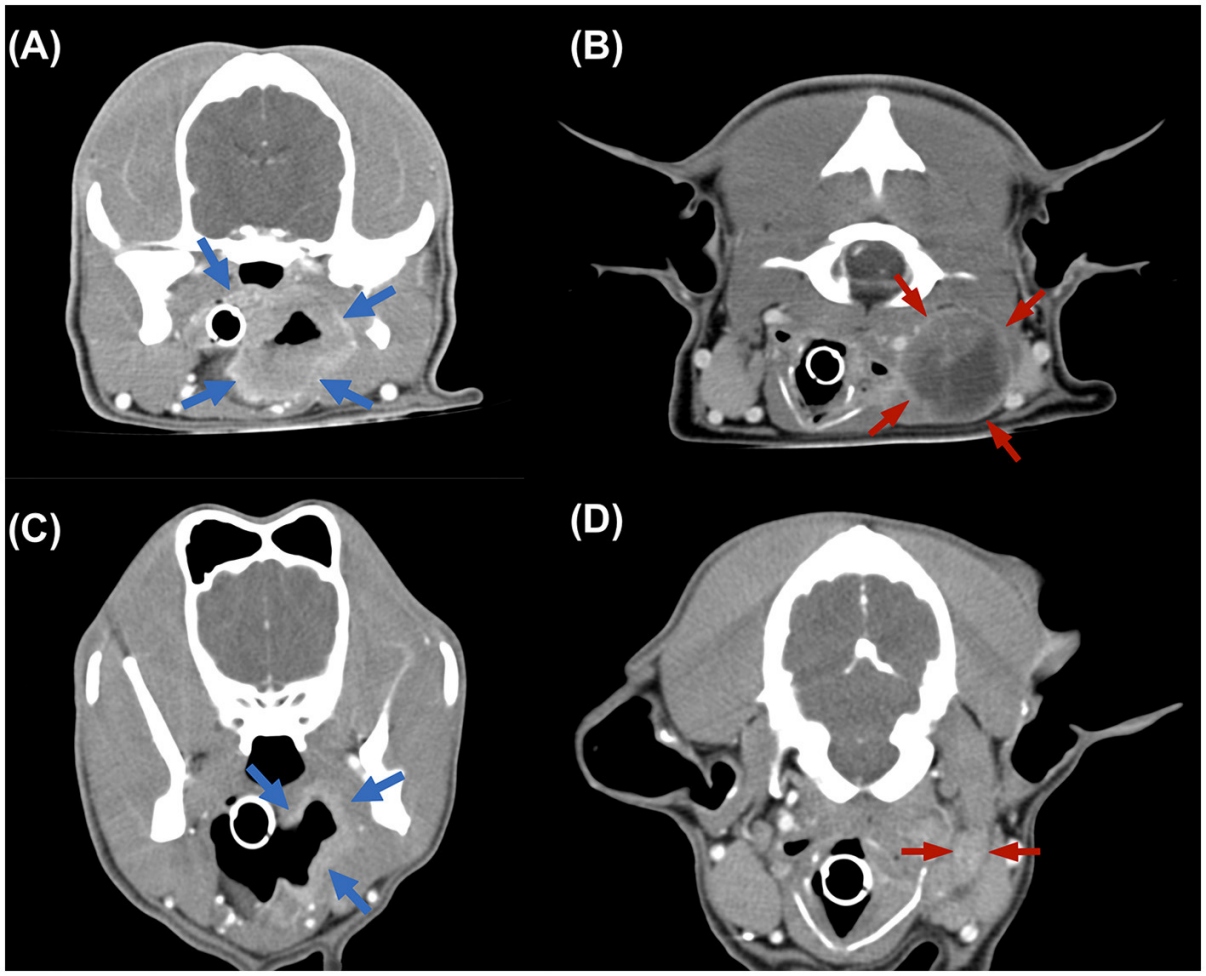
Transverse computed tomography (CT) images. **(A, B)** CT images acquired before treatment, revealing the irregular mass from pharynx (blue arrow) and the enlarged medial retropharyngeal lymph node, 3.88 × 3.01 × 3.12 cm in size (red arrow). **(C, D)** Images from follow-up 4 months CT examination revealing the mass and LN both reached partial remission.

Then the oral examination and computed tomography (CT) scan was performed under general anesthesia. The oral examination revealed a ring-thickened mass on the left soft palate (Figure 1A). And CT scan further disclosed this pharyngeal mass as irregular in shape, 6.93 × 3.63 × 2.94 cm in size, characterized by ring and heterogeneous contrast enhancement with slight enhancement in the inner band and significant enhancement in the outer band, involving soft palate, epiglottis, left tonsil, part of the cricoid cartilage and adjacent muscles such as left medial pterygoid muscle, left digastric muscle and initiation of the longus capitis muscle (Figure 2A). In addition, the left medial retropharyngeal LN was irregularly enlarged with necrosis (3.88 × 3.01 × 3.12 cm; Figure 2B). And a mild enlargement of left mandibular LN was also found (1.74 × 0.66 cm). Without evidence of distant metastasis, the main differential diagnosis included salivary gland carcinoma, tonsil squamous cell carcinoma, OMM, fibrosarcoma, sarcoma of muscular origin.

**Figure 3:**
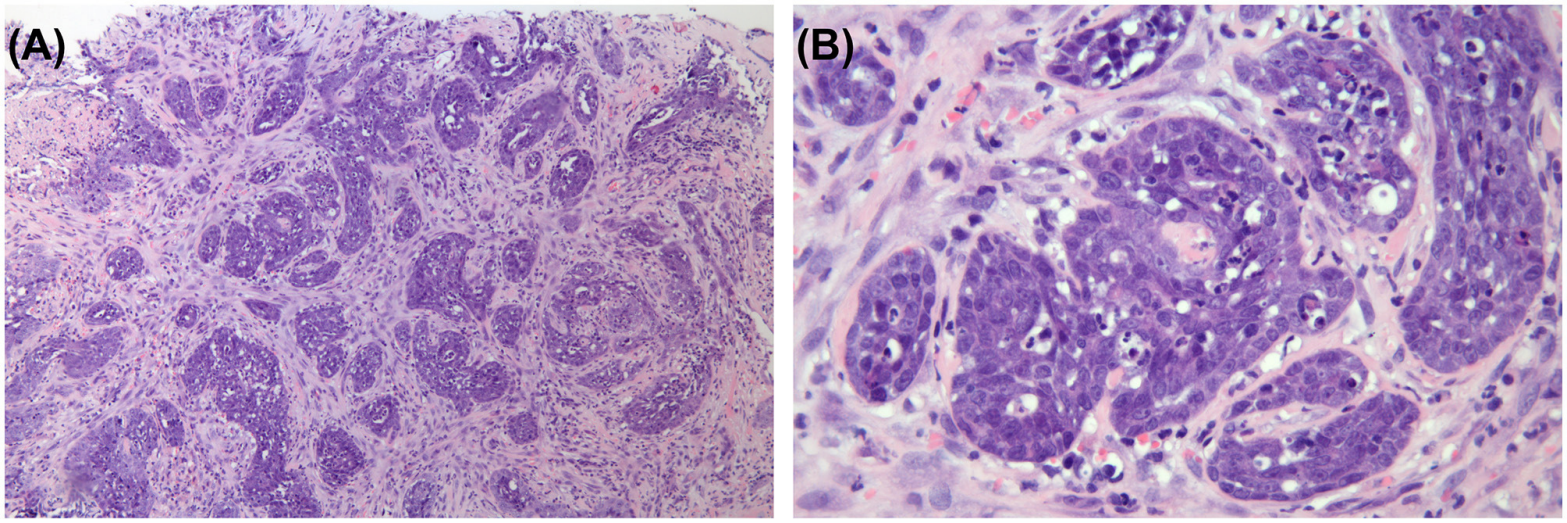
Histopathological examination of the mass. Neoplastic mass consisted of long, dense, irregularly interlaced bundles of spindle cells, and a few of them formed irregular alveoli. Hematoxylin and eosin stain. **(A)** ×100. **(B)** ×400.

Fine needle aspiration (FNA) cytology from soft palate and incisional biopsy of the mass was performed. Cytology was suspicious of malignancy and the specific origin could not be distinguished. Sections of the mass were processed for histology, and embedded in paraffin. Sections (5 μm) were cut and stained with hematoxylin and eosin (Figure 3). Histologically, the neoplasm is made up of pleomorphic columnar to round cells arranged in trabeculae and nests, occasionally formed irregular alveoli. The neoplastic cells have indistinct cell borders, small to moderate amounts of eosinophilic cytoplasm, and a round to ovoid nuclei with coarsely clumped chromatin and 1–3 prominent nucleolus. There is marked anisocytosis and anisokaryosis. Mitotic rate is 4 in 10 high-power fields corresponding to 2.37 mm^2^.

**Figure 4:**
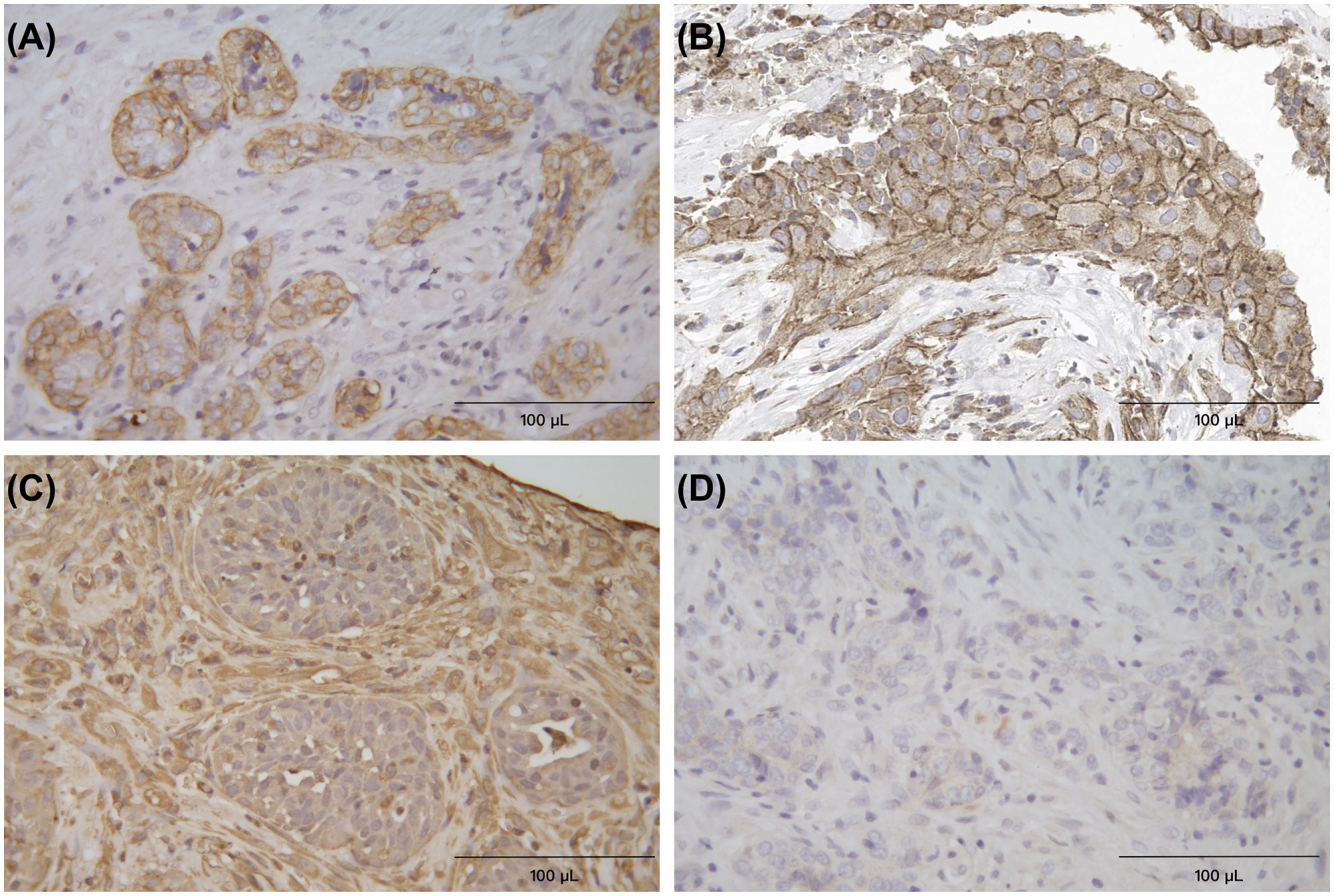
Immunohistochemical (IHC) staining for cytokeratin **(A)**, PD-L1 **(B)**, vimentin **(C)**, and melan-A **(D)**, respectively, the Horseradish Peroxidase (HRP) method, neoplastic cells formed the glandular appearance and were intensely positive on cytokeratin and PD-L1, and negative on vimentin and melan-A. The surrounding connective tissue cells were positive on vimentin ×400.

Additional sections were used for immunohistochemical (IHC) staining (using the Horseradish Peroxidase method), with antibodies against cytokeratin (clone AE1/AE3 + 5D3; 1:200 dilution; Abcam, Cambridge, England), vimentin (clone V9; 1:200 dilution; Abcam), and melan-A (clone A103; 1:200 dilution; Dakocytomation, Glostrup, Denmark). Neoplastic cells were intensely positive on cytokeratin IHC, and they were mostly negative on vimentin and melan-A IHC (Figure 4). Histological and immunohistochemical results were consistent with a diagnosis of adenocarcinoma, most likely originating from the minor salivary gland. What's more, according to the WHO modified tumor node metastases (TNM) system (12), this case was stage III oral adenocarcinoma. In order to better indicate the treatment of anti-PD-1, PD-L1 IHC (Anti-PD-L1 Recombinant Rabbit Monoclonal Antibody; Huabio, Hangzhou, China) was performed on tumor cells, and the results were cytoplasmic positive (Figure 4B).

Due to the difficulty of the surgery and the inability to complete excision, the owner agreed to join the PD-1 clinical trial, and the first dose was started on the 8^th^ day after the first presentation. Based on information from other previous studies (10, 11) and several preclinical trials, we used caninized anti-PD-1 monoclonal antibody MP001 at 3 mg/kg dose. MP001 was dissolved using normal saline and administered intravenously, every 2 weeks.

CBC and serum biochemistry follow-up identified no abnormalities after first two doses. However, the patient suddenly developed dyspnea right before the third dose. The sign gradually resolved after oxygen therapy, and examination proceeded, with no abnormality detected by follow-up CBC, serum biochemistry, and two-view thoracic radiographs examination. Cervical ultrasound suggested the mass had processed to about 5.12 × 2.98 × 5.79 cm in size which is about 3.5 times larger than the size at diagnosis, and the left medial retropharyngeal LN also enlarged (4.53 × 1.98 cm). Then before the fifth dose treatment, follow-up ultrasound suggested the mass reached partial remission (4.24 × 3.33 × 1.90 cm, reduced the volume by 70%) and the LN was significantly smaller (thickness of 0.43 cm) compared that to images from a month ago. Four months after the diagnosis, before the eighth dose, the gross appearance of the tumor was significantly reduced (Figure 1B). Follow-up CT re-examination demonstrated that the pharyngeal mass had almost disappeared and the left medial retropharyngeal LN was only slightly enlarged (Figures 2C, D), with no evidence of distant metastasis.

In addition to dyspnea, gastrointestinal adverse events including vomiting and diarrhea only observed before the fifth treatment. And these adverse events are grade 1, according to VCOG-CTCAE v1.1 (13). No other adverse events were observed.

At 6-month follow-up after CT re-examination, the patient remained in partial remission without metastasis. But the patient was euthanized later due to severe osteoarthritis of the hip. The survival time is 316 days and duration of response (DOR) is 252 days.

## 3. Discussion

The case was eventually diagnosed as oral adenocarcinoma. Based on anatomic location and IHC staining, we think this mass was originated from minor salivary gland of pharynx or soft palate. Adenocarcinoma of minor salivary gland is very rare in dogs, but should be included in the differential diagnosis list of oral tumors. The median age of dogs with salivary gland tumors is 10.5-year-old (1). Surgery is the mainstay of therapy. Postoperative radiotherapy is usually recommended, especially if the tumor is aggressive and has outgrown the capsule (14). A median survival time (MST) of 550 days was reported in 24 dogs with salivary gland adenocarcinomas in one study, but compared with stage I and II, dog on stage III and IV had significantly shorter survival times of <100 days (2).

This case was stage III according to a WHO modified tumor node metastases (TNM) system (12). Partial remission was reached after the fourth dose and DOR exceeded 8 months. These fully indicate that PD-1 blockade reverses PD-1-mediated anti-tumor immunosuppression and effectively inhibits tumor progression.

Adverse events from immunotherapy are usually self-limiting. Compared with chemotherapy or molecular targeted therapy, ICI therapy has the advantage of causing fewer adverse events. The most common adverse events include fatigue, fever, anorexia, and gastrointestinal signs such as vomiting and diarrhea (10), which are usually grade 1–2 according to VCOG-CTCAE v1.1 (13). In this case, the patient had diarrhea and vomiting events during treatment period, which gradually improved without intervention. The gastrointestinal symptoms presented were grade 1, and were suspected to be due to food change, based on history and gastrointestinal ultrasound results. The only exception was the dyspnea developed before the third dose, which was a grade 2 adverse event.

Surprisingly, after the fourth dose, the mass reached partial remission. Therefore, we hypothesized that the enlargement of the mass after the second dose was pseudoprogression, which is kind of unconventional responses to anti PD-1 therapy. Pseudoprogression is defined as an initial increase in tumor growth followed by a decrease (15). It is not true tumor progression, because the actual volume of the tumor parenchyma does not increase. These changes can be observed by tumor biopsy or continuous radiation scan (16). Once the inflammation subsides, the tumor responds positively to treatment. Pseudoprogression may be caused by reactivation of the immune system, leading to an influx of immune cells into the tumor microenvironment and a transient increase in inflammation and tumor burden (17). Unfortunately, in this case, we did not perform any procedures to verify pseudoprogression, and could only suspect the existence of such unconventional responses based on imaging changes. However, this is the first time that pseudoprogression has been observed in dogs treated with ICI in our hospital, and provides more information for future clinical trials. The examination should be refined in future cases.

Antibodies that block PD-1 signaling can reverse PD-L1 mediated suppression of T-cell activation (18, 19). Therefore, we can indirectly and grossly assess the degree of T cell inhibition by the expression level of PD-L1 in tumor cells. Many studies have confirmed the expression of PD-L1 in different canine cancers, including anal sac gland carcinoma, hemangiosarcoma, nasal adenocarcinoma, transitional cell carcinoma, osteosarcoma, OMM, mast cell tumor, lymphoma and so on (11, 20, 21).

In this case, we confirmed the expression of PD-L1 in tumor cells using IHC (Figure 4B), indicating that PD-L1 in tumor cells may bind to PD-1 in immune cells and confirming that PD-1 antibody can act through the PD-1 signaling pathway. However, there is no absolute correlation between the expression of PD-L1 and the efficacy of immunotherapy, and some studies have even found that PD-1 treatment can still be effective even if the expression of PD-L1 is negative (10). More research is needed to explore the mechanism of anti-PD-1 therapy in dogs.

In summary, a canine salivary gland carcinoma was effectively treated using canine PD-1 monoclonal antibody. Both survival time and DOR were longer than that in previous studies, confirming the great potential of ICI therapy. As the trial progresses, more information should be provided to veterinarians about ICI treatment, including but not limited to the applicable tumor type, possible side effects, and factors affecting ICI treatment.

## Data availability statement

The raw data supporting the conclusions of this article will be made available by the authors, without undue reservation.

## Ethics statement

The animal study was reviewed and approved by Ethic Committee of China Agricultural University Laboratory Animal Welfare and Animal Experiment (Issue No. Aw40103202-2-1). Written informed consent was obtained from the participant/patient(s) for the publication of this case report.

## Author contributions

SX wrote the manuscript. GL assisted in supervision of the clinical management of this case and contributed to conception of the case report. JX created the PD-1 antibodies for clinical trials. JF and NT supervised the clinical management of this case. SW and GL revised the manuscript. YS assisted in the histopathological staining of the PD-L1. All authors critically reviewed and approved the final version of the manuscript.

## References

[1] Cray M Selmic LE Ruple A. Salivary neoplasia in dogs and cats: 1996–2017. Vet Med Sci. (2020) 6:259–64. 10.1002/vms3.228 31849188 PMC7397883

[2] Hammer A Getzy D Ogilvie G Upton M Klausner J Kisseberth W. Salivary gland neoplasia in the dog and cat: survival times and prognostic factors. J Am Anim Hosp Assoc. (2001) 37:478–82. 10.5326/15473317-37-5-478 11563448

[3] Hodi FS O'Day SJ McDermott DF Weber RW Sosman JA Haanen JB. Improved Survival with Ipilimumab in Patients with Metastatic Melanoma. N Engl J Med. (2010) 363:711–23. 10.1056/NEJMoa1003466 20525992 PMC3549297

[4] Gordon SR Maute RL Dulken BW Hutter G George BM McCracken MN. PD-1 expression by tumour-associated macrophages inhibits phagocytosis and tumour immunity. Nature. (2017) 545:495–9. 10.1038/nature22396 28514441 PMC5931375

[5] Pesce S Greppi M Grossi F Del Zotto G Moretta L Sivori S. PD/1-PD-Ls checkpoint: insight on the potential role of NK cells. Front Immunol. (2019) 10:1242. 10.3389/fimmu.2019.01242 31214193 PMC6557993

[6] Baumeister SH Freeman GJ Dranoff G Sharpe AH. Coinhibitory pathways in immunotherapy for cancer. Annu Rev Immunol. (2016) 34:539–73. 10.1146/annurev-immunol-032414-112049 26927206

[7] Freeman GJ Long AJ Iwai Y Bourque K Chernova T Nishimura H. Engagement of the PD-1 immunoinhibitory receptor by a novel B7 family member leads to negative regulation of lymphocyte activation. J Exp Med. (2000) 192:1027–34. 10.1084/jem.192.7.1027 11015443 PMC2193311

[8] Latchman Y Wood CR Chernova T Chaudhary D Borde M Chernova I. PD-L2 is a second ligand for PD-1 and inhibits T cell activation. Nat Immunol. (2001) 2:261–8. 10.1038/85330 11224527

[9] Twomey JD Zhang B. Cancer Immunotherapy Update: FDA-Approved Checkpoint Inhibitors and Companion Diagnostics. AAPS J. (2021) 23:39. 10.1208/s12248-021-00574-0 33677681 PMC7937597

[10] Igase M Nemoto Y Itamoto K Tani K Nakaichi M Sakurai M. A pilot clinical study of the therapeutic antibody against canine PD-1 for advanced spontaneous cancers in dogs. Sci Rep. (2020) 10:18311. 10.1038/s41598-020-75533-4 33110170 PMC7591904

[11] Maekawa N Konnai S Nishimura M Kagawa Y Takagi S Hosoya K. PD-L1 immunohistochemistry for canine cancers and clinical benefit of anti-PD-L1 antibody in dogs with pulmonary metastatic oral malignant melanoma. NPJ Precis Oncol. (2021) 5:10. 10.1038/s41698-021-00147-6 33580183 PMC7881100

[12] Seifert G Brocheriou C Cardesa A Eveson JW WHO. International Histological Classification of Tumours. Tentative Histological Classification of Salivary Gland Tumours. Pathol Res Pract. (1990) 186:555–81. 10.1016/S0344-0338(11)80220-7 1962854

[13] Veterinary cooperative oncology group - common terminology criteria for adverse events. VCOG-CTCAE following chemotherapy or biological antineoplastic therapy in dogs and cats v1.1. Vet Comp Oncol. (2016) 14:417–46. 10.1111/vco.283 28530307

[14] Evans SM Thrall DE. Postoperative orthovoltage radiation therapy of parotid salivary gland adenocarcinoma in three dogs. J Am Vet Med Assoc. (1983) 182:993–4. 6853325

[15] Chiou VL Burotto M. Pseudoprogression and Immune-Related Response in Solid Tumors. J Clin Oncol. (2015) 33:3541–3. 10.1200/JCO.2015.61.6870 26261262 PMC4622096

[16] Reckamp KL. Real-World Pseudoprogression: an Uncommon Phenomenon. J Thorac Oncol. (2018) 13:880–2. 10.1016/j.jtho.2018.05.011 29935844

[17] Li N Wang G Hou X Tai R Huang S He Z. Adverse and unconventional reactions related to immune checkpoint inhibitor therapy for cancer. Int Immunopharmacol. (2022) 108:108803. 10.1016/j.intimp.2022.108803 35569432

[18] Coy J Caldwell A Chow L Guth A Dow S. PD-1 expression by canine T cells and functional effects of PD-1 blockade. Vet Comp Oncol. (2017) 15:1487–502. 10.1111/vco.12294 28120417

[19] Maekawa N Konnai S Ikebuchi R Okagawa T Adachi M Takagi S. Expression of PD-L1 on canine tumor cells and enhancement of IFN-γ production from tumor-infiltrating cells by PD-L1 blockade. PLoS ONE. (2014) 9:e98415. 10.1371/journal.pone.0098415 24915569 PMC4051644

[20] Shosu K Sakurai M Inoue K Nakagawa T Sakai H Morimoto M. Programmed cell death ligand 1 expression in canine Cancer. Vivo Athens Greece. (2016) 30:195–204. 27107075

[21] Maekawa N Konnai S Okagawa T Nishimori A Ikebuchi R Izumi Y. Immunohistochemical Analysis of PD-L1 expression in canine malignant cancers and PD-1 expression on lymphocytes in canine oral melanoma. PLoS ONE. (2016) 11:e0157176. 10.1371/journal.pone.0157176 27276060 PMC4898770

